# Influenza and Other Respiratory Viruses Detected by Influenza-Like Illness Surveillance in Leyte Island, the Philippines, 2010–2013

**DOI:** 10.1371/journal.pone.0123755

**Published:** 2015-04-20

**Authors:** Hirono Otomaru, Taro Kamigaki, Raita Tamaki, Jamie Opinion, Arlene Santo, Edgard Daya, Michiko Okamoto, Mariko Saito, Veronica Tallo, Soccoro Lupisan, Akira Suzuki, Hitoshi Oshitani

**Affiliations:** 1 Department of Virology, Tohoku University School of Medicine, Sendai, Japan; 2 Tacloban City Health Office, Tacloban City, the Philippines; 3 Tanauan Rural Health Unit, Tanauan, the Philippines; 4 Leyte Provincial Health Office, Palo, the Philippines; 5 Tohoku-RITM Research Center for Emerging and Reemerging Infections, Muntinlupa City, the Philippines; 6 Research Institute for Tropical Medicine, Muntinlupa City, the Philippines; 7 Virus Research Center, Clinical Research Division, Sendai Medical Center, Sendai, Japan; Centers for Disease Control, TAIWAN

## Abstract

This study aimed to determine the role of influenza-like illness (ILI) surveillance conducted on Leyte Island, the Philippines, including involvement of other respiratory viruses, from 2010 to 2013. ILI surveillance was conducted from January 2010 to March 2013 with 3 sentinel sites located in Tacloban city, Palo and Tanauan of Leyte Island. ILI was defined as fever ≥38°C or feverish feeling and either cough or running nose in a patient of any age. Influenza virus and other 5 respiratory viruses were searched. A total of 5,550 ILI cases visited the 3 sites and specimens were collected from 2,031 (36.6%) cases. Among the cases sampled, 1,637 (75.6%) were children aged <5 years. 874 (43.0%) cases were positive for at least one of the respiratory viruses tested. Influenza virus and respiratory syncytial virus (RSV) were predominantly detected (both were 25.7%) followed by human rhinovirus (HRV) (17.5%). The age distributions were significantly different between those who were positive for influenza, HRV, and RSV. ILI cases were reported throughout the year and influenza virus was co-detected with those viruses on approximately half of the weeks of study period (RSV in 60.5% and HRV 47.4%). In terms of clinical manifestations, only the rates of headache and sore throat were significantly higher in influenza positive cases than cases positive to other viruses. In conclusion, syndromic ILI surveillance in this area is difficult to detect the start of influenza epidemic without laboratory confirmation which requires huge resources. Age was an important factor that affected positive rates of influenza and other respiratory viruses. Involvement of older age children may be useful to detect influenza more effectively.

## Introduction

Influenza is an acute viral respiratory infection that affects 5%–15% of the global population and leads to approximately 250,000–500,000 deaths annually worldwide [1]. Because of the public health significance, many countries have established their own influenza surveillance programs. Influenza surveillance is conducted for different objectives, including monitoring of circulating viruses, antiviral drug susceptibility, trends and impact of seasonal epidemics, and emergence of viruses with a pandemic potential. This surveillance provides useful information to both public health authorities and medical professionals. Recently, the newly emergent influenza A (H7N9) virus was reported in China, and influenza surveillance was able to detect and monitor the outbreak [2]. Influenza-like illness (ILI), often defined as a sudden onset of fever >38°C and cough and/or sore throat, is a syndrome that is widely used for monitoring influenza in outpatient settings. The positive predictive values (PPVs) and negative predictive values (NPVs) of the case definitions were 52% and 82%, respectively, in a cohort study in Taiwan during the early phase of pandemic (H1N1) 2009 [3] and 77% and 51%, respectively, in a multicenter study in temperate regions for seasonal influenza [4]. PPV could be as low as 36% if the study period covered both epidemic and non-epidemic periods [5]. In addition, ILI surveillance is commonly used to monitor influenza epidemiology. Previous studies have revealed that the detection rates of influenza range from 8.2% to 36.6% [6–10].

In temperate regions, there is a clear seasonality of influenza epidemics with the peaks during wintertime while in tropic region, that clear seasonal pattern is not usually observed [11]. In the Philippines, influenza virus was detected throughout the year from both outpatients and inpatients [12]. A certain level of sensitivity is required to monitor the trend of seasonal influenza and to detect viruses with a pandemic potential. In order to determine the role of ILI surveillance in the tropical area, we conducted the ILI surveillance with virological examination in Leyte Island, the Philippines, including involvement of other respiratory viruses, from 2010 to 2013.

## Materials and Methods

### Study sites

**Fig 1:**
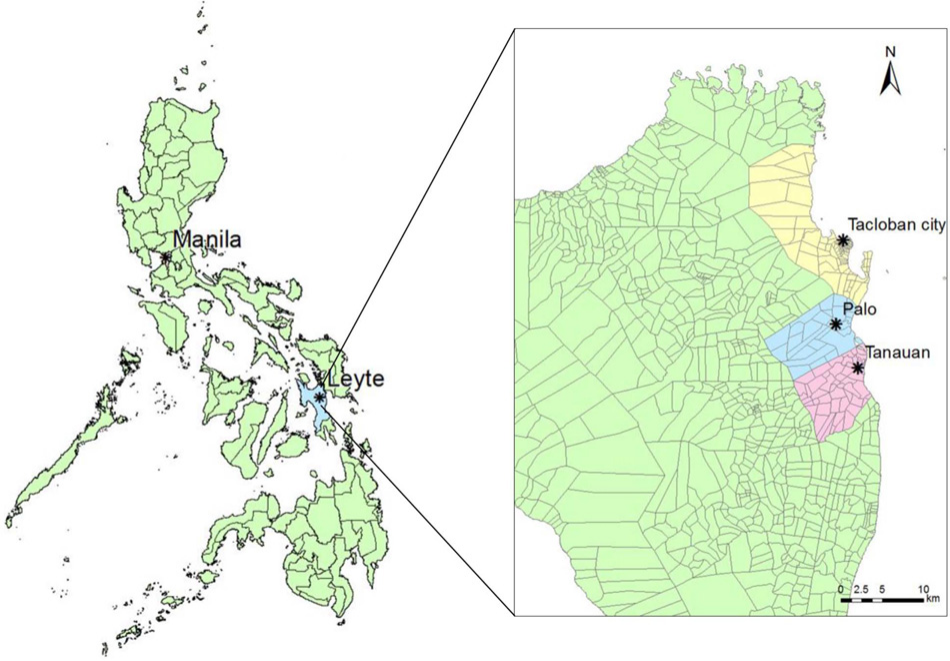
Locations of the 3 sentinel sites in the Eastern Visayas region, the Philippines. Map source; DIVA-GIS website (http://www.diva-gis.org/gdata).

The Philippines is located mostly in a tropical region and consists of 17 administrative regions. The Eastern Visayas region is located in the central eastern part of the country. Tacloban city is the capital city of the region, which is located approximately 580 km southeast of Manila. The total population of this region is approximately 4.1 million. ILI surveillance was conducted from January 2010 to March 2013 at 3 sentinel sites located in Tacloban city and the neighboring municipalities of Palo and Tanauan. According to the 2010 national census, approximately 221,200 people live in Tacloban city, 62,700 in Palo, and 50,100 in Tanauan [13]. The 3 sentinel sites included Tacloban City Health Center (T-CHC, Tacloban city), Leyte Provincial Hospital (LPH, Palo), and Tanauan Rural Health Unit (T-RHU, Tanauan) (Fig 1). T-CHC and T-RHU are health centers and LPH is a hospital with a 50-bed capacity.

### Case definitions and sample collection

Patients with ILI who visited the 3 health facilities from January 2010 to March 2013 were asked to participate in the study. ILI was defined as fever ≥38°C or feverish feeling and either cough or running nose in a patient of any age with refer to WHO guideline [14]. Sampling was conducted on 1 or 2 days per week at each sentinel site because of limited resources. Patients with ILI who visited the sentinel sites on the sampling day were asked to undergo nasopharyngeal or throat swabbing if the interval from the date of onset was ≤5 days. The collected specimens were placed in 3 ml of viral transport medium containing Hank’s balanced salt solution (Sigma-Aldrich, St. Louis, Mo., USA)) with 0.25% gelatin, 500 units/ml penicillin, 500 μg/ml streptomycin, and 0.5 μg/ml fungizone and were stored at 4°C–10°C until they were transported to RITM for processing.

### Laboratory testing

In molecular virological testing, RNA and DNA were extracted from the sample swabs using the QIAamp MinElute Virus Spin kit (Qiagen, Hilden, Germany). To synthesize complementary DNA, 11 μl of the final extract was used as the template with M-MLV, random hexamers, and RNase inhibitor (all from Invitrogen Carlsbad, CA, USA). Monoplex realtime polymerase chain reaction (PCR) for influenza A virus using the Taqman real-time PCR system (Applied Biosystems, Carlsbad, USA), and monoplex PCR was performed to screen for influenza B virus, human rhinovirus (HRV), human adenovirus (HAdV), and parainfluenza virus (PIV) using ExTaq (TaKaRa Bio, Otsu, Japan) [15–19]. Multiplex real-time PCR was performed to screen for respiratory syncytial virus (RSV) [20] and human metapneumovirus (hMPV) with same real-time PCR system for influenza A virus screening [21]. Negative and positive controls were included in each run.

In virus isolation testing, viruses were isolated using a microplate method with 6 cell lines; Madin—Darby canine kidney (MDCK), human laryngeal carcinoma (HEp-2), African green monkey kidney (VeroE6), human embryonic lung fibroblast (HFL), habdomyosarcoma (RD-A), and green monkey kidney (GMK) cells. [22–24]. HFL cell line was bought from RIKEN Bioresourse center CELL BANK (Tsukuba, Japan) and others were offered from Sendai Medical Center. The inoculated cells were observed for cytopathic effects (CPEs). For identifying strain of influenza, hemagglutination inhibition (HI) assay using antibodies (Denka seiken, Tokyo, Japan) were also performed when specific CPE was observed on MDCK cell and hemagglutination assay (HA) positive with guinea pig erythrocytes. Antibodies were targeted for the vaccine strain for northern hemisphere which is specified by WHO. When the subtype of influenza could not be identified by HI assay, confirmatory PCR were performed using same system as molecular virological testing section for detecting novel influenza strain. Influenza C virus was also detected by HA assay using turkey erythrocytes and confirmatory PCR were performed [19]. Influenza C virus causes agglutination in the chicken or turkey erythrocytes, but does not cause agglutination of the guinea pig cells. For other viruses, confirmatory PCR was performed for the target virus when typical CPEs were observed.

### Data collection and statistical analysis

A standardized questionnaire was used to collect necessary information from all ILI consulted cases. The questionnaire included demographic information, consultation date, and presenting clinical manifestations. All data were encoded into a database. Frequencies of epidemiological and clinical variables as well as the set of respiratory viruses were counted. The Mann—Whitney *U*-test and Kruskal—Wallis rank test were used for continuous variables. The chi-square test or Fisher’s exact test was used for categorical variables such as the clinical characteristics of the patients. A *p*-value of <0.05 was considered significant. All tests were conducted with IBM SPSS Statistics 20 (IBM Corp., Armonk, NY, USA) or R. 3.0.2 (R Foundation, Vienna, Austria).

### Ethics statement

This study was conducted as a component of ILI national surveillance under the Philippine Integrated Disease Surveillance and Response (PIDSR) program. For children aged <12 years, written informed consent for participation in the study was obtained from their parents or guardians. For children aged 12–18 years, written informed consent/assent was required prior to the collection of epidemiological and clinical information in addition to the parent or guardian’s consent. In case of patients aged >18 years, written consent was obtained from the participants themselves. The study protocol was approved by the ethical review board of Tohoku University Graduate School of Medicine (ID 2014-1-334).

## Results

The demographic characteristics of the ILI cases are presented in Table 1. A total of 5,550 ILI cases visited the 3 sites during the study period. In total, 5,143 (92.7%) cases met the sampling criteria, and specimens were collected from 2,031 (36.6%) cases. The percentage of cases sampled to total ILI cases was highest in 2013 and the differences between the years were significant (*p* < 0.001). Among the cases sampled, 960 (47.3%) were male, and 1,637 (75.6%) were children aged <5 years. The male-to-female ratio was not significantly different, whereas the proportion of samples tested by age groups as well as by sentinel sites were significantly different (chi-square test, *p <* 0.001 for both).

**Table 1:** Demographic characteristics of all influenza-like illness (ILI) cases and the tested cases from 2010 to 2013.

	Covariate	Total ILI consultations	No. of tested cases (%)	*p*-value
Year	2010	1657	599 (36.1)	<0.001
	2011	1929	632 (32.8)	
	2012	1723	655 (38.0)	
	2013	241	145 (60.2)	
Age	0–5 months	549	168 (30.6)	<0.001
	6–11 months	843	328 (38.9)	
	1–4 years	2699	1039 (38.5)	
	5–9 years	924	341 (36.9)	
	10–14 years	288	112 (38.9)	
	15–19 years	61	15 (24.6)	
	20–39 years	116	21 (18.1)	
	Over 40 years	70	7 (10.0)	
Sex	Female	2835	1071 (37.8)	0.07
	Male	2715	960 (35.4)	
Facility	LPH	2146	752 (35.0)	<0.001
	T-CHC	2040	613 (30.0)	
	T-RHU	1364	666 (48.8)	

Abbreviations: LPH, Leyte Provincial Hospital; T-CHC, Tacloban City Health Center; T-RHU, Tacloban Rural Health Unit.

As shown in Table 2, 874 (43.0%) cases were positive for at least 1 of the respiratory viruses tested. A total of 225 influenza virus-positive cases were observed, and the overall positive rate for influenza viruses was 11.1%. Of the influenza positive cases, 52.0% were influenza A, 46.2% were influenza B, and 1.8% were influenza C. Among the influenza A positive cases, 55 (47.0%) were influenza A (H1N1) pdm09 and 61 (52.1%) were influenza A (H3N2). Only 1 seasonal influenza A (H1N1) case was detected in 2010. RSV was detected in 225 (11.1%) cases, followed by HRV (7.5%) and hMPV (3.6%). Other viruses, including HAdV, PIV, herpesvirus, cytomegarovirus, and enteroviruses accounted for 197 (9.7%) cases. The details of the other viruses detected are shown in S1 Table.

**Table 2:** Viral etiology for influenza-like illness (ILI) cases from 2010 to 2013.

			Subtotal (%)	2010 (%)	2011 (%)	2012 (%)	2013* (%)
**No. of samples**			2031	599	632	655	145
**Influenza**			225 (11.1)	96 (16.0)	92 (14.6)	21 (3.2)	16 (11.0)
	Influenza A		117 (52.0)	44 (45.8)	55 (59.8)	16 (7.6)	2 (12.5)
		A (H1N1) pdm09	55 (47.0)	15 (34.1)	40 (72.7)	0 (0.0)	0 (0.0)
		Seasonal A (H1N1)	1 (0.9)	1 (2.3)	0 (0.0)	0 (0.0)	0 (0.0)
		A (H3N2)	61 (52.1)	28 (63.6)	15 (27.3)	16 (100)	2 (100)
	Influenza B		104 (46.2)	52 (54.2)	34 (37.0)	5 (23.8)	13 (81.3)
	Influenza C		4 (1.8)	0 (0.0)	3 (3.3)	0 (0.0)	1 (6.3)
**RSV**			225 (11.1)	49 (8.2)	83 (13.1)	61 (9.3)	32 (22.1)
**HRV**			153 (7.5)	72 (12.0)	53 (8.4)	14 (2.1)	14 (9.7)
**hMPV**			74 (3.6)	15 (2.5)	19 (3.0)	38 (5.8)	2 (1.4)
**Others**			197 (9.7)	40 (6.7)	70 (11.1)	77 (11.8)	10 (6.9)

* In 2013, samples were collected until March.

Abbreviations: RSV, respiratory syncytial virus; HRV, human rhinovirus; hMPV, human metapneumovirus.

The age distribution of cases that were positive for influenza viruses, RSV, HRV, and hMPV is summarized in Table 3. The highest positive rates for influenza viruses were observed in the age group of >5 years (18.5%–46.7%), except the age group of >40 years. RSV positive rates were higher among children aged <5 years (12.9%–16.7%). Higher HRV positive rates were observed in infants, and the values were higher than those for influenza. The age distributions were significantly different between those who were positive for influenza, HRV, and RSV (Kruskal—Wallis test, *p* < 0.01), whereas they were not significantly different among those who were positive for influenza viruses and subtypes (*p* = 0.52). The number of virus-positive cases among adults was small for all tested viruses. Only a single case aged >40 years was positive for HRV. This was partly because the number of adult consultations was much smaller than the number for children aged <5 years.

**Table 3:** Aggregate number of virus positives and positivity rates (%) by age group from 2010 to 2013.

	Total tested	Influenza	A (H1N1) pdm09	Seasonal A (H1N1)	A (H3N2)	Influenza B	Influenza C	RSV	HRV	hMPV
**0–5 months**	168	9 (5.4)	3 (1.8)	0	2 (1.2)	3 (1.8)	1 (0.6)	28 (16.7)	19 (11.3)	3 (1.8)
**6–11 months**	328	22 (6.7)	4 (1.2)	1 (0.1)	11 (3.4)	5 (1.5)	2 (0.6)	49 (14.9)	38 (11.6)	16 (4.9)
**1–4 years**	1039	93 (9.0)	22 (2.1)	0	28 (2.7)	41 (3.9)	1 (0.1)	134 (12.9)	67 (6.4)	44 (4.2)
**5–9 years**	341	63 (18.5)	17 (5.0)	0	11 (3.2)	35 (10.3)	0	9 (2.6)	17 (5.0)	9 (2.6)
**10–14 years**	112	26 (23.2)	6 (5.4)	0	5 (4.5)	15 (13.4)	0	4 (3.6)	6 (5.4)	2 (1.8)
**15–19 years**	15	7 (46.7)	1 (6.7)	0	1 (6.7)	5 (33.3)	0	1 (6.7)	1 (6.7)	0
**20–39 years**	21	5 (23.8)	2 (9.5)	0	3 (14.3)	0	0	0	4 (19.0)	0
**Over 40 years**	7	0	0	0	0	0	0	0	1 (14.3)	0

Abbreviations: RSV, respiratory syncytial virus; HRV, human rhinovirus; hMPV, human metapneumovirus.

**Fig 2:**
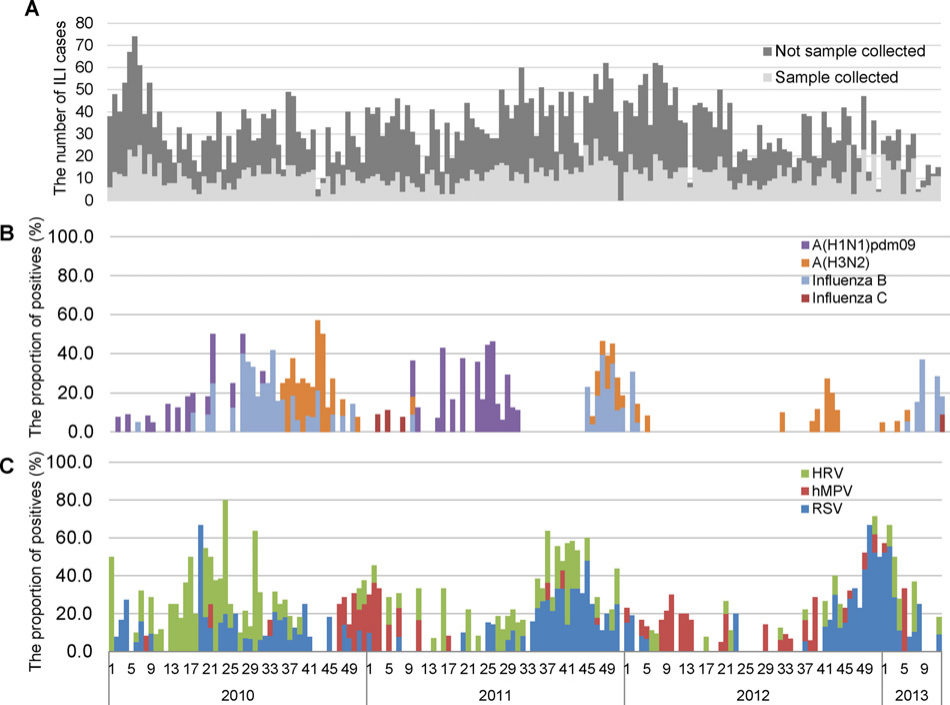
A–C Weekly number of influenza-like illness (ILI) (A), and weekly proportions of influenza positives (B), and virus positives other than influenza (C) from 2010 to 2013. Abbreviations: RSV, respiratory syncytial virus; HRV, human rhinovirus; hMPV, human metapneumovirus

The weekly number of total ILI consultations and of samples collected are shown in Figs 2A and S1. During the entire study period, the mean percentage of samples collected per week was 39.2%. ILI cases were reported throughout the year and did not show any clear seasonal pattern. The weekly percentages of influenza-, RSV-, HRV-, and hMPV-positive cases are shown in Fig 2B and 2C. Influenza A (H3N2) and influenza B cases were detected every year during the study period. Both viruses were circulating during the same period in 2010 and 2011; however, they were not co-circulating in 2012 and 2013. Influenza A (H1N1) pdm09 was detected in 2010 and 2011. The peaks of each influenza virus did not overlap. RSV showed rather an annual cyclic pattern, with peaks in week 45 of 2011 and week 51 of 2012; however, only sporadic cases were detected in 2010. During the 168 morbidity weeks studied, there were 76 weeks when ≥1 influenza virus was detected per week; the results for the other viruses are as follows: 82 weeks for RSV, 73 weeks for HRV, and 43 weeks for hMPV. Furthermore, influenza virus was co-detected with at least 1 of the other 3 viruses in 80.3% of the 76 weeks (60.5% for RSV, 47.4% for HRV, and 19.7% for hMPV). Therefore, other viruses were also detected with influenza during most of the study period.

**Fig 3:**
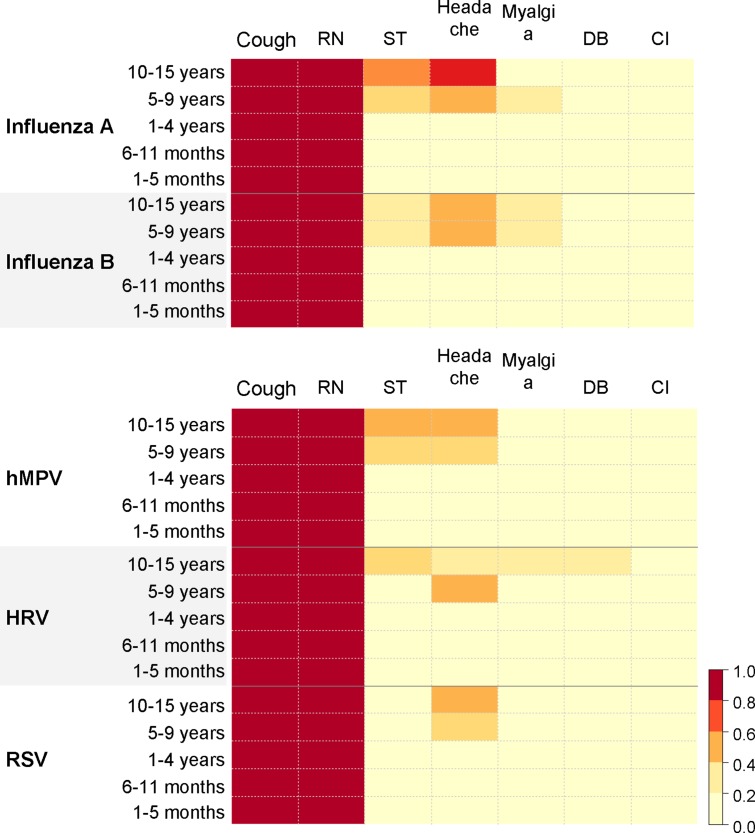
Frequencies of clinical manifestations presented among cases aged up to 15 years. Abbreviations: RN, runny nose; ST, sore throat; DB, difficulty in breathing; CI, chest indrawing, hMPV, human metapneumovirus; HRV, human rhinovirus; RSV, respiratory syncytial virus.

Fig 3 maps the frequencies of clinical manifestations presented at the time of consultation for each virus-positive case by age group up to 15 years. Both cough and runny nose were observed in nearly 100% of cases for all 5 viruses, as these manifestations were included in our case definitions. Besides of these symptoms, significantly higher rates of headache and sore throat were observed in cases of influenza than in cases of other viruses (*p* = 0.009 and 0.01 respectively) by age group up to 15 years; however, headache was observed in only 26.2% of influenza cases (S2 Table). No significant differences were observed in the rates of muscle ache or difficulty in breathing (*p* = 0.14, 0.21 respectively), but the rate of chest indrawing was higher in RSV-positive cases than in other cases (*p* = 0.04). All symptoms studied were observed ≥20% more frequently among children aged >5 years.

## Discussion

The purpose of influenza surveillance includes to monitor circulating influenza viruses, describe influenza seasonality, and to establish the baseline of activity for influenza then ILI surveillance is most effective when combing the laboratory testing [25]. In our study, over 70% of ILI cases seen at the 3 sentinel sites were children aged <5 years. Other studies in developing countries have also reported a high proportion of young children among ILI cases [26,27]. This result can be explained by differences in health-seeking behavior between adults and children, i.e., young children with ILI tend to visit healthcare facilities more often than adults. The health-seeking behavior of residents was not investigated at our study sites. However, the fact that the proportion of older children and adults were smaller than young children in the proportion of ILI count might partly reflect unwilling to come to the hospital. More information not only health seeking behavior but also the absent count of school children or ILI case count for medication store for adults might be helpful.

The overall positive rate for at least 1 respiratory virus was 43.0%. This may be attributable to a lower virus titer at the time of sample collection, sample quality, and other causative agents not tested in this study. However, our data revealed the epidemiological characteristics of influenza and other respiratory viruses on Leyte Island, the Philippines. Among the cases who were positive for at least 1 virus, influenza and RSV accounted for 25.7%, followed by HRV (17.5%), and hMPV (8.5%). This result suggests that the case definitions of ILI detect not only influenza but also other virus infections, particularly RSV and HRV infections. Influenza viruses were one of the most common viral agents in the tested samples, similar to the results reported previously [8,27–29]. RSV was another commonly occurring virus in this study. The rates of RSV positives among children aged < 5 years were reported as 28.2% in Cote d’Ivoire [9] and 13% in Belgium [30]. Thus, RSV is one of the leading viral agents among younger children. Some studies enrolling all-aged patients have detected HRV as frequently as influenza [27,31]. The rate of RSV-positive cases among at least 1 virus-positive case varied between years, ranging from 18.0% to 28.9%, and the rate for HRV varied from 6.6% to 26.5% in 2010–2013. The previous etiological study of respiratory viruses among pediatric hospitalized cases in a referral hospital in this study area conducted from 2008 to 2009 found that the most common virus detected was HRV followed by RSV and that both RSV and HRV were much more common than influenza viruses [32].

Compared with RSV- and HRV-positive cases, the age distribution of influenza-positive cases was concentrated to older age groups. This trend was compatible with findings documented in other studies. A study of patients with acute respiratory illness in the 2009–2010 seasons in Japan showed that the ratio of RSV decreased with age [33]. A study among children with lower respiratory tract infection in Egypt showed that the ratio of influenza increased with age [34]. A Chinese study of outpatients with ILI showed that the ratio of RSV-positive cases aged >3 years to those aged <1 year was 0.31, while those ratios among HRV-, and influenza- positive cases [excluding A(H1N1)pdm09] were 1.72 and 2.47, respectively [35]. The differences of those ratios were comparable to estimates in a study by Cote d'Ivoire [9]. Thus, age can be an important factor to improve sensitivity of targeting etiologic viruses, particularly among children.

The overlap of clinical manifestations between influenza and other respiratory virus infections has been clearly documented [36]. Both sore throat and headache were significantly higher in influenza cases, but these manifestations were mainly seen in older children. Headache is typically reported by adults [37], and only a minority of younger children with influenza present with headache [38]. Inclusion of these symptoms in the case definitions of ILI can increase the sensitivity of the case definition if a higher proportion of ILI cases are observed among older children such as school-aged children who are able to report their subjective manifestations. However, in our setting, >70% of children with ILI visiting healthcare facilities were aged <5 years, and there was little advantage of increasing the likelihood of detecting influenza virus by changing the combination of clinical symptoms beyond our initial set of symptoms.

Influenza B co-circulated with influenza A (H3N2) in 2010 and 2011 with different peak timings. The earlier peak of influenza B before A (H3N2) in 2010 and the delayed peak in 2011 compared with 2010 has been documented in other countries such as the United States [28] and Hong Kong [39]. In addition, those studies also documented fewer influenza A (H1N1) pdm09 cases after 2012. Overall, influenza virus accounted for 11.1% of ILI cases, and we did not observe clear influenza seasonality in the study area. Less clear seasonality has also been documented in other tropical and subtropical countries [11,40]. Further monitoring is necessary to define influenza seasonality in the Philippines. Influenza and other viruses were co-detected in approximately 80% of the weeks in this study. Previous studies in Hong Kong [39] and Japan [41] have shown that the RSV peak overlapped or preceded the peak of the influenza epidemic. Other reports from the United Kingdom [42] and Israel [43] showed that RSV activity preceded or overlapped with hMPV activity. In this study, influenza activity overlapped with RSV activity, and RSV was observed during the preceding or overlapping period of hMPV circulation. Thus, ILI activity consisted of various activities of multiple respiratory viruses in addition to the less clear seasonal pattern of influenza virus. This suggested that the increase of ILI does not always indicate the influenza epidemic particularly when most ILI cases were small children.

In summary, it is difficult to follow influenza activity only by means of syndromic ILI surveillance because of the less clear seasonality of influenza in the study site and partly because of the low specificity for detecting influenza infections. There are several possible reasons for such low specificity. First, many ILI cases in this study were small children who were more likely to be infected with other viruses such as RSV and hMPV. Much more investigation about other age group might be helpful for detection of influenza activity. Second, small children usually do not complain of some influenza-specific symptoms such as headache. Third, the seasonality of influenza in this region is less clear same as other tropical area because influenza were detected all year round. Fourth, influenza detection was overlapped with other respiratory viruses so that simply monitoring frequency of ILI without laboratory confirmation were not enough to follow influenza epidemic start.

This study had some limitations. First, there was a potential selection bias in the ILI cases due to the different health-seeking behavior of the different age group, which may have affected the observed importance of viral etiology. Second, we could not test all samples because of limited resources, and sampling was not necessarily systematic; this may have affected our interpretation of respiratory virus epidemiology. Third, our surveillance was implemented at 3 sentinel sites in Tacloban city and its surrounding areas, which limit the capability to generalize our findings to the entire country. Fourth, >50% of the samples were not positive for any of the tested viruses, and other etiological agents, including bacterial pathogens, were not tested in this study.

## Conclusions

In conclusion, we studied the clinical and virological features of ILI cases in Leyte Island, the Philippines. ILI surveillance detected influenza virus as a leading agent but seasonality of influenza was unclear throughout study period. The detection of other respiratory viruses were quite overlapped and the clinical characteristics of influenza were also quite similar to those of other respiratory viruses particularly in small children. Therefore, syndromic ILI surveillance in this area is difficult to grasp the trend of influenza epidemic without laboratory confirmation which requires a huge resources. Age was an important factor that affected positive rates of influenza and other respiratory viruses. Including cases of older age groups may be useful to detect influenza more effectively. Further studies are necessary to establish the monitor of influenza activities through ILI surveillance program more economically in developing tropical countries where influenza virus does not show clear seasonality and where most ILI cases visiting healthcare facilities are small children.

## Supporting Information

S1 Fig Weekly number of ILI consultations and proportion of samples collected from 2010 to 2013. Abbreviations: ILI, influenza like illness.
(TIF)

S1 Table The number of multiple virus positive cases and the number of positive cases with parainfluenza virus, adenovius, herpesvirus, cytomegalovirus, and enterovirus. (DOCX)

S2 Table Frequencies of clinical manifestations for influenza A (subtype), influenza B, influenza C, RSV, HRV, and hMPV by age groups. Abbreviations: RSV, respiratory syncytial virus; HRV, human rhinovirus; hMPV, human metapneumovirus.
(DOCX)
