# Supplementary figures and images for: Influenza and Other Respiratory Viruses Detected by Influenza-Like Illness Surveillance in Leyte Island, the Philippines, 2010–2013
Source: PLoS One. 2015 Apr 20;10(4):e0123755. doi: 10.1371/journal.pone.0123755 (PMC4404362; doi:10.1371/journal.pone.0123755)

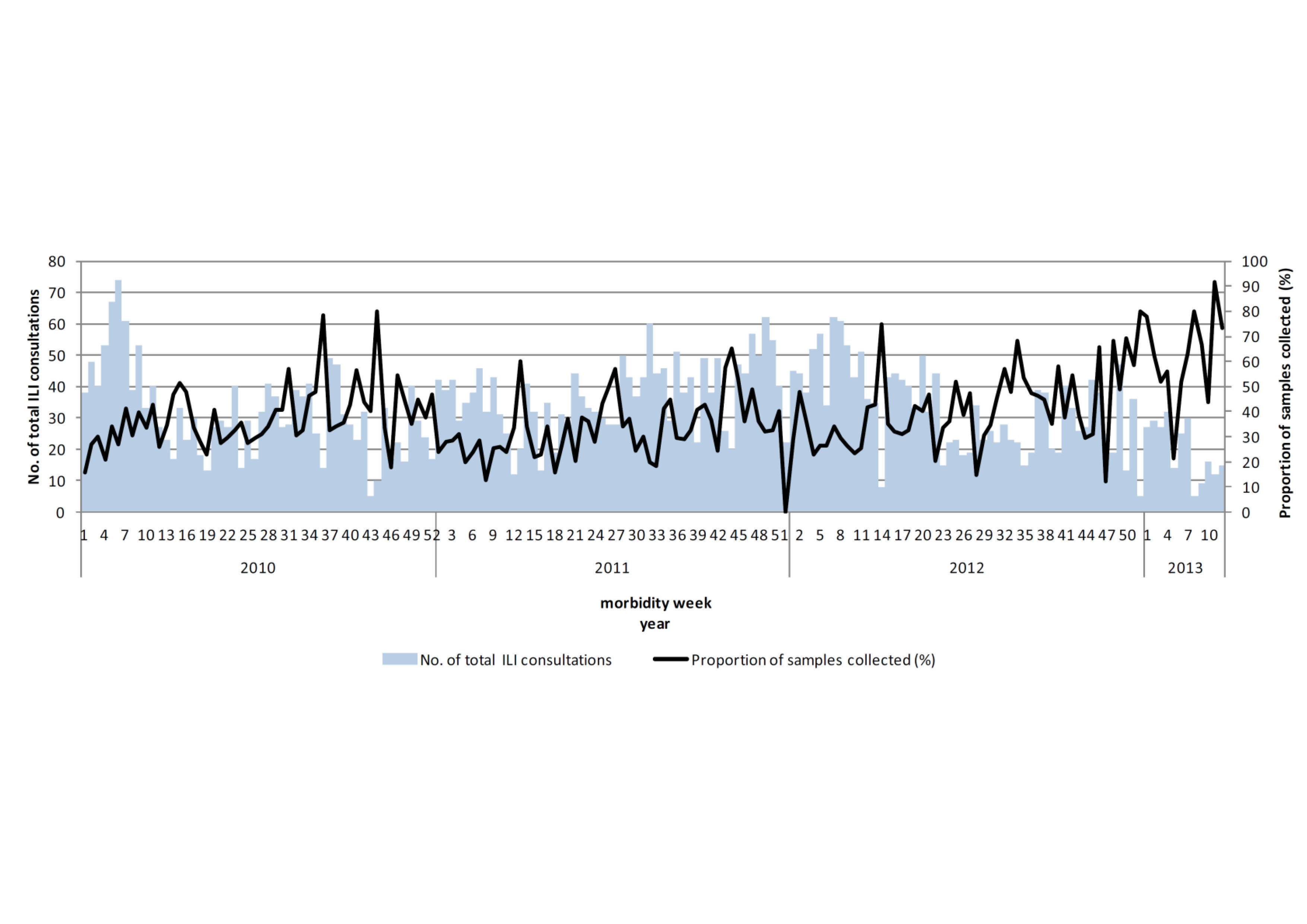

Supplement: S1 Fig — Abbreviations: ILI, influenza like illness. (TIF) [file pone.0123755.s001.tif]
