# Supplementary material for: Influenza and Other Respiratory Viruses Detected by Influenza-Like Illness Surveillance in Leyte Island, the Philippines, 2010–2013
Source: PLoS One. 2015 Apr 20;10(4):e0123755. doi: 10.1371/journal.pone.0123755 (PMC4404362; doi:10.1371/journal.pone.0123755)
Supplement: S1 Table — (DOCX) [file pone.0123755.s002.docx]

S1 Table. The number of multiple virus positive cases and the number of positive cases with parainfluenza virus, adenovius, herpesvirus, cytomegalovirus, and enterovirus

| **Viral etiology** | **No. of cases** | **Detection method** |
| --- | --- | --- |
| Single detection | 145 |  |
| Parainfluenza virus | 49 (33.8) | PCR |
| Adenovirus | 48 (33.1) | PCR |
| Herpesvirus | 14 (9.7) | Virus isolation |
| Cytomegalovirus | 12 (8.3) | Virus isolation |
| Enterovirus | 22 (15.2) | Virus isolation |
| Multiple detection |  |  |
| 2 viruses | 51 |  |
| 3 viruses | 1 |  |
