# Supplementary material for: Influenza and Other Respiratory Viruses Detected by Influenza-Like Illness Surveillance in Leyte Island, the Philippines, 2010–2013
Source: PLoS One. 2015 Apr 20;10(4):e0123755. doi: 10.1371/journal.pone.0123755 (PMC4404362; doi:10.1371/journal.pone.0123755)
Supplement: S2 Table — Abbreviations: RSV, respiratory syncytial virus; HRV, human rhinovirus; hMPV, human metapneumovirus. (DOCX) [file pone.0123755.s003.docx]

S2 Table. Frequencies of clinical manifestations for influenza A (subtype), influenza B, influenza C, RSV, HRV, and hMPV by age groups

|  |  | Cough | Runny nose | Sore throat | Headache | Muscle ache | Difficulty in breathing | Chest indrawing |
| --- | --- | --- | --- | --- | --- | --- | --- | --- |
| <2 years | Total | 2461 | 2429 | 12 | 11 | 1 | 69 | 57 |
|  | FluA (H1N1) pdm09 | 13 | 13 | 0 | 0 | 0 | 0 | 0 |
|  | FluA (H1N1) | 1 | 1 | 0 | 0 | 0 | 0 | 0 |
|  | FluA (H3N2) | 25 | 25 | 0 | 0 | 0 | 0 | 0 |
|  | FluB | 18 | 18 | 0 | 0 | 0 | 0 | 0 |
|  | FluC | 3 | 3 | 0 | 0 | 0 | 0 | 0 |
|  | RSV | 137 | 137 | 1 | 1 | 1 | 2 | 4 |
|  | HRV | 84 | 83 | 0 | 0 | 0 | 3 | 2 |
|  | hMPV | 37 | 36 | 0 | 0 | 0 | 1 | 2 |
| 2–5 years | Total | 1857 | 1802 | 90 | 237 | 17 | 30 | 18 |
|  | FluA (H1N1) pdm09 | 20 | 20 | 1 | 4 | 0 | 1 | 0 |
|  | FluA (H3N2) | 18 | 18 | 0 | 0 | 0 | 1 | 0 |
|  | FluB | 38 | 38 | 1 | 3 | 1 | 0 | 0 |
|  | FluC | 1 | 1 | 0 | 0 | 0 | 0 | 0 |
|  | RSV | 78 | 77 | 0 | 5 | 1 | 2 | 2 |
|  | HRV | 43 | 43 | 2 | 5 | 0 | 2 | 0 |
|  | hMPV | 30 | 30 | 1 | 3 | 0 | 0 | 0 |
| 6–10 years | Total | 738 | 699 | 247 | 339 | 75 | 25 | 0 |
|  | FluA (H1N1) pdm09 | 16 | 16 | 6 | 8 | 2 | 0 | 0 |
|  | FluA (H3N2) | 13 | 12 | 8 | 8 | 3 | 1 | 0 |
|  | FluB | 31 | 30 | 8 | 15 | 4 | 0 | 0 |
|  | RSV | 7 | 7 | 0 | 2 | 0 | 1 | 0 |
|  | HRV | 16 | 16 | 2 | 4 | 1 | 2 | 0 |
|  | hMPV | 6 | 6 | 3 | 4 | 0 | 0 | 0 |
|  | Total | 205 | 183 | 76 | 113 | 31 | 16 | 1 |
| 11–15 | FluA (H1N1) pdm09 | 3 | 3 | 1 | 3 | 0 | 0 | 0 |
| years | FluA (H3N2) | 1 | 1 | 0 | 1 | 0 | 0 | 0 |
|  | FluB | 15 | 15 | 2 | 9 | 4 | 1 | 0 |
|  | RSV | 2 | 2 | 0 | 1 | 0 | 0 | 0 |
|  | HRV | 3 | 3 | 1 | 1 | 0 | 0 | 0 |
|  | hMPV | 1 | 1 | 0 | 0 | 0 | 0 | 0 |

Abbreviations: FluA, influenza A virus; FluB, influenza B virus; FluC, influenza C virus; RSV, respiratory syncytial virus; HRV, human rhinovirus; hMPV, human metapneumovirus
